# Allergic predisposition modifies the effects of pet exposure on respiratory disease in boys and girls: the seven northeast cities of china (snecc) study

**DOI:** 10.1186/1476-069X-11-50

**Published:** 2012-07-23

**Authors:** Guang-Hui Dong, Jing Wang, Miao-Miao Liu, Da Wang, Yungling Leo Lee, Ya-Dong Zhao

**Affiliations:** 1 Department of Biostatistics and Epidemiology, School of Public Health, China Medical University, 92 North 2nd Road, Heping District, Shenyang, Liaoning Province, 110001, China; 2 Department of Occupational and Environmental Health, School of Public Health, China Medical University, Shenyang, Liaoning Province, 110001, China; 3 Department of Biostatistics, School of Public Health, Saint Louis University, Saint Louis, MO, 63104, USA; 4 Institute of Epidemiology and Preventive Medicine, College of Public Health, National Taiwan University, Taipei, 100, Taiwan; 5 Institute of Respiratory Diseases, the First Affiliated Hospital of China Medical University, Shenyang, Liaoning Province, 110001, China

**Keywords:** Pet exposure, Asthma, Allergic Predisposition

## Abstract

**Background:**

The relationship between pet exposure and the respiratory disease in childhood has been a controversial topic, much is still unknown about the nature of the associations between pet exposure and children’s respiratory health stratified by gender and allergic predisposition. The objective of the present study was to assess the relationship between pet exposure and respiratory symptoms in Chinese children, and to investigate the modified effects of gender and allergic predisposition on such relationship.

**Methods:**

31,049 children were selected from 25 districts of 7 cities in Northeast China in 2009. Information on respiratory health and exposure to home environmental factors was obtained *via* a standard questionnaire designed by the American Thoracic Society.

**Results:**

Children with an allergic predisposition were found to have more frequent exposure to pets than those without an allergic predisposition (18.5% *vs.* 15.4%). In children without an allergic predisposition, pet exposure was associated with increased susceptibility to respiratory symptoms/diseases, with girls being more susceptible than boys. No association was found between pet exposure and respiratory symptoms/diseases in boys with an allergic predisposition. In girls with an allergic predisposition, association was found between doctor-diagnosed asthma and pet exposure of their mother during pregnancy (adjusted odds ratio (ORs) = 2.03; 95% confidence interval (CI): 1.01-4.33), and their current pet exposure (ORs = 1.37; 95%CI: 1.00-1.88).

**Conclusions:**

Pet exposure in children without an allergic predisposition was associated with increased susceptibility to respiratory disease, with girls being more susceptible than boys.

## Background

The effects of pet exposure on the development of respiratory symptoms have been a controversial topic [1-6]. The SIDRIA-2 study in Italy reported that exposure to pets in children in the first year of life was a significant and independent risk factor for current asthma and asthma related symptoms that appeared at the age of 7 [1]. Results from the International Study of Asthma and Allergy in Childhood (ISAAC) showed a positive association between pet exposure during pregnancy and in the first year of life and asthma, eczema and wheeze in 6 - to 7-year-old children [2]. Further analysis showed that this positive association was more evident in children in developing countries than in developed countries [3]. However, other studies showed that pet exposure in children during the first year of life could provide a protective effect against the development of asthma, allergic rhinitis and eczema in later life [4-6]. A systematic review concluded that exposure to pets increases the risk of asthma and wheezing only in children older than 6 years of age [7], whereas studies from British and Germany showed that the risk of asthma and asthma related symptoms caused by pet exposure was relative low among children older than 8 years of age and among adults [8,9]. Recent studies suggested that the seemingly protective effect of pet exposure may be a result of a “healthy pet keeping effect”, in that parents with asthmatic diseases tend to keep their child from being exposed to pets to protect them against childhood asthma [10-12].

The association of pet exposure with childhood asthma seems to vary globally, perhaps because children with asthma tend to become sensitized to the allergens prominent in their living environments [9]. The association of race with allergic sensitization among children seems to also vary across different countries [3,13,14]. For example, Hugg *et al.* compared the relationships between pet exposures and the occurrence of allergic asthma in Finnish and Russian school children. Their results indicated that the risk of allergic asthma was inversely related to indoor pet-keeping in Finland, whereas in Russia the risk of allergic asthma increased in relation to indoor pet exposure [14]. Also, recently, the Phase Three of the International Study of Asthma and Allergies in Childhood (ISAAC) study reported that pet exposure was associated with increased symptoms of asthma only in children living in non-affluent [3]. However, to the best of our knowledge, few studies have evaluated the health effects of pet exposure in Chinese children, and few studies have considered the modification effect of allergic predisposition in evaluation of the associations between pet exposure and respiratory symptoms. Also, there has been growing epidemiologic evidence of a difference association between environmental factors and respiratory health between boys and girls [15-18], so it is plausible that boys and girls may respond differently to pet exposure as well. Pet exposure was quite prevalent in China due to the lack of knowledge or awareness of the risk factors for asthma and allergies and the lack of primary prevention strategies. This may produce “clean” data that alleviates bias from occurring and, therefore, allows for close examination of the effect of pet exposure on respiratory symptoms among children. We conducted a cross-sectional survey on children from 7 cities in Northeast China, and investigated the relationship between pet exposure and respiratory symptoms/diseases in Chinese children, and to investigate the modified effects of gender and allergic predisposition on such relationship in this study population.

## Methods

### Participants and study procedures

The seven northeast cities of China (SNECC) study was designed using the guidelines set by the Ethical Standards of Responsible Committee on Human Experimentation of China Medical University. Seven cities (Shenyang, Dalian, Anshan, Fushun, Benxi, Liaoyang and Yingkou) were randomly selected from Liaoning Province in April of 2009. The numbers of districts in these 7 cities were, respectively, 5 in Shenyang, 4 in Dalian and Fushun, and 3 in Anshan, Benxi, Liaoyang and Yingkou. Two kindergartens and one elementary school were randomly selected from each district, resulting in a total of 50 kindergartens and 25 elementary schools, and all their students were given a questionnaire and return envelope. After parents/guardians reviewed the questionnaire, we invited them to a Parents’ Night and explained to them detailed information about the survey, including the objective of the survey and a revocable parental consent form, asking for parent’s permission for their child’s voluntary participation in the survey. Parents or guardians who wished to complete the questionnaire at home would have their child return the completed questionnaire in an envelope to the teacher. To reduce biased results, we explained to the parents/guardians and their children that the purpose of the survey was to study the relationship between respiratory health and general environmental factors, without specifying pet exposure.

### Questionnaire data

We assessed children’s respiratory health and potential risk factors, including demographics and pet-keeping, *via* a questionnaire that consisted of a few respiratory health related questions from the American Thoracic Society Epidemiologic Standardization Project Questionnaire in Chinese translation [19,20], which has been proved to be an effective assessment tool in a few studies [19-21].

### Definitions of respiratory symptoms and illnesses

The following respiratory symptoms and illnesses were determined from the questionnaire responses: a) Doctor diagnosed asthma, defined as a positive answer to the question “Has a doctor ever diagnosed asthma in this child?”; b) Current asthma: defined as, for the child who had been diagnosed with asthma, a positive answer to the question “Has this child been in a paroxysm of asthma in the last two years?” or a positive answer to the question “Has this child every taken medicine or treatment for asthma or asthmatic bronchitis?”; c) Current wheeze, defined as a positive answer to the question “Has this child’s chest ever sounded wheezy or whistling, including times when he or she had a cold?” and a positive answer to the question “Has this child had 2 or more such episodes in the last 12 months?”; d) Persistent cough, defined as ever having cough for more than 4 days per week for at least 3 months, either with or without cold, during the 12 months prior to the assessment; e) Persistent phlegm, defined as ever having been congested or ever having phlegm, sputum, or mucus brought up from the chest for more than 4 days per week for at least 3 months, either with or without a cold, during the 12 months prior to the assessment.

Family history of allergies was defined as a family history of doctor-diagnosed hay fever or allergies (including allergic dermatitis, allergic conjunctivitis, and eczema). Family history of asthma was defined as a family history of doctor-diagnosed asthma or bronchial asthma. Allergic predisposition was defined as a family history of allergy or asthma. Personal allergic history was defined as a family history of allergic constitution, allergic rhinitis or atopic eczema, hay fever, allergies to food or medicine, inhaled dusts, pollen, molds, animal fur or dander, or skin allergies (without the inclusion of allergy to poison ivy or oak).

Current pet exposure was assessed *via* questions on the type and number of animals kept in the household during the past 12 months. A dummy variable was created with 2 levels (1 = Yes, 0 = No (the reference category)), depending on whether or not the child had ever been exposed to dogs, cats, farm animals or other types of animals such as chickens, ducks, cows and pigs. We also assessed pet exposure of the mother during pregnancy and their child during the first year of life. Possible pet avoidance measures for pet allergies were assessed *via* 2 questions: “Has your family given up a pet due to allergies in the family?” and “Has your family avoided getting a pet due to allergies in the family?”

### Statistical analysis

We fitted a multivariate logistic regression model using each of the 5 outcome measures of respiratory symptoms and the predictor variables including pet exposure and all other covariates. From the fitted model, we selected the covariates that had a positive association with the respiratory symptoms/diseases. Using these selected covariates, we carried out backward selection by dropping the variable that had a less than 10% decrease in the model fit statistic after the variable was removed from the model. The final model calculated the adjusted odds ratios (ORs) and the associated 95% confidence intervals (CI) for each outcome measure (SAS 9.13, SAS Institute, Inc., Cary, NC, USA). All statistical tests were two-tailed and a p-value less than 0.05 was considered statistically significant.

## Results

Among the total of 35,527 children from 50 preschools and 25 elementary schools, 31,049 completed and returned the questionnaire, yielding an overall response rate of 87.4%; among the respondents, 15,673 (50.5%) were males. The participation rates varied from 81.3% in Yingkou to 94.7% in Dalian, which did not correlated with either levels of pet exposure or diseases prevalence. The mean age of the participants was 8.5 with a standard deviation (SD) 2.7 (range of ages: 2.2 - 13.4 years). Allergic predisposition was observed in 13.7% of the participants. Participants with a pet at home had a higher rate of allergic predisposition than those without pets at home (18.5% *vs.* 15.4%). The characteristics of the participants and conditions of exposure to pets, parental atopy, and other risk factors in the home environment are shown in Table 1. Boys appeared to be more likely to have respiratory symptoms and asthma, exposure to pet during pregnancy, and have commercial health insurance than girls.

**Table 1:** Demographics of the study population of boys (N = 15,673) and girls (N = 15,376) in seven cities of Northeast China

**Characteristic**	**Boys (n, %)**	**Girls (n, %)**	**Total (n, %)**
*Asthma and asthma related symptoms*			
Doctor-diagnosed asthma	1179 (7.5)	856 (5.6)^*^	2035 (6.6)
Current asthma	410 (2.6)	288 (1.9)^*^	698 (2.3)
Current wheeze	1082 (6.9)	883 (5.7)^*^	1965 (6.3)
Persistent cough	1538 (9.8)	1439 (9.4)	2977 (9.6)
Persistent phlegm	761 (4.9)	660 (4.3)^*^	1421 (4.6)
*Allergic predisposition*			
Family history of allergic	1124 (7.2)	1153 (7.5)	2277 (7.3)
Family history of asthma	1227 (7.8)	1185 (7.7)	2412 (7.8)
Family history of allergic/asthma	2134 (13.6)	2106 (13.7)	4240 (13.7)
Personal allergic history	3393 (21.7)	3006 (19.6)^*^	6399 (20.6)
Personal hay fever history	2101 (13.4)	1368 (8.9)^*^	3469 (11.2)
*Environmental factors exposures*			
Exposure to pets during pregnancy	627 (4.0)	523 (3.4)^*^	1150 (3.7)
Exposure to pets in the first years of life	878 (5.6)	968 (6.3)	1846 (6.0)
Avoidance to pet exposure	141 (0.9)	108 (0.7)	249 (0.8)
*Current exposure to pets*			
Yes	2339 (14.9)	2569(16.7)^*^	4908(15.8)
*Number of pets*			
1	1886 (12.0)	2027 (13.2)	3913 (12.6)
≥2	453 (2.9)	542 (3.5)	995 (3.2)
Dogs	878 (5.6)	999 (6.5)^*^	1877 (6.0)
Cats	736 (4.7)	830 (5.4)^*^	1566 (5.0)
Birds	455 (2.9)	507 (3.3)	962 (3.1)
Farm animals	282 (1.8)	384 (2.5)^*^	666 (2.1)
Other pets	533 (3.4)	523 (3.4)	1056 (3.4)
ETS exposure^†^	7779 (49.6)	7537 (49.0)	15316 (49.3)
House decoration in recent two years	5342 (34.1)	5223 (34.0)	10565 (34.0)
Breast feeding	13517 (86.2)	13464 (87.6)^*^	26981 (86.9)
Number of room <3	8484 (54.1)	8183 (53.2)	16667 (53.7)
Home coal use	1055 (6.7)	1016 (6.6)^*^	2071 (6.7)
Commercial health insurance	4059 (25.9)	2260 (14.7)^*^	6319 (20.4)
Education level of parents < high school	4361 (27.8)	4123 (26.8)^*^	8484 (27.3)

^†^*ETS,* Environmental tobacco smoke. ^*^The difference between boys and girls is significant at the 0.05 level.

Logistic regression analyses were performed to evaluate the potential confounding effects of allergic predisposition on the relationship between pet exposure and respiratory outcomes. In general, children without an allergic predisposition were found to have greater values of ORs than those with an allergic predisposition (Table 2). For example, significant associations were observed for doctor-diagnosed asthma with pet exposure of the mother during pregnancy (OR =1.58, 95%CI: 1.12-2.24), pet exposure of the child in the first year of life (OR =1.59, 95%CI: 1.19-2.13), and current pet exposure of the child (OR =1.46, 95%CI: 1.27-1.67) only in children without an allergic predisposition. Associations between current asthma and pet exposure during pregnancy (OR =2.92, 95%CI: 1.84-4.62), pet exposure in the first year of life (OR =3.05, 95%CI: 2.05-4.53) were noticeably strong. In children without an allergic predisposition, more pets were associated with a higher rate of all respiratory symptoms/diseases except current asthma (*e.g.*, for persistent cough: OR = 1.31, 95%CI: 1.03-1.67 for 1 pet and OR = 1.66, 95%CI: 1.48-1.86 for more than 1 pet), and for doctor-diagnosed asthma and current wheeze, significance was only observed for more than 1 pet (OR =1.54, 95%CI: 1.34-1.78 for doctor-diagnosed asthma; OR =1.31, 95%CI: 1.12-1.53 for current wheeze). Among the children with an allergic predisposition, significant associations were mainly observed between pet exposure and persistent cough, persistent phlegm. Even for the persistent cough and persistent phlegm, most of the adjusted ORs among subjects without allergic predisposition were higher than those among subjects with allergic predisposition. For doctor-diagnosed asthma, current asthma, and current wheeze, children without an allergic predisposition in general had bigger values of ORs than children with an allergic predisposition. We therefore speculate that children without an allergic predisposition in general may be more susceptible to these symptoms/diseases than children with an allergic predisposition.

**Table 2:** Association of children’s respiratory symptoms and diseases with pet exposure in children with and without allergic predisposition^*^

**Characteristics**	**Persistent cough**	**Persistent phlegm**	**Doctor-diagnosed asthma**	**Current asthma**	**Current wheeze**
***Children without allergic predisposition (n = 26,809)***					
Exposure to pets during pregnancy (ref: no)	1.09 (0.79-1.50)	**1.53 (1.04-2.26)**	**1.58 (1.12-2.24)**	**2.92 (1.84-4.62)**	**2.27 (1.62-3.17)**
Exposure to pets in the first years (ref: no)	1.11 (0.85-1.44)	1.10 (0.76-1.59)	**1.59 (1.19-2.13)**	**3.05 (2.05-4.53)**	**2.31 (1.74-3.07)**
Current exposure to pets (ref: no) †	**1.60 (1.43-1.78)**	**1.89 (1.64-2.19)**	**1.46 (1.27-1.67)**	1.16 (0.90-1.50)	**1.29 (1.12-1.50)**
Number and type of pets†					
1	**1.31 (1.03-1.67)**	**1.73 (1.27-2.36)**	1.27 (0.91-1.76)	1.27 (0.97-1.66)	1.23 (0.89-1.53)
≥2	**1.66 (1.48-1.86)**	**1.93 (1.65-2.25)**	**1.54 (1.34-1.78)**	0.67 (0.33-1.37)	**1.31 (1.12-1.53)**
Cats	**1.79 (1.49-2.14)**	**2.35 (1.88-2.95)**	**1.68 (1.34-2.10)**	**1.48 (1.00-2.22)**	**1.37 (1.06-1.78)**
Dogs	**1.25 (1.03-1.51)**	**1.32 (1.01-1.73)**	**1.25 (1.00-1.58)**	0.79 (0.47-1.32)	1.22 (0.95-1.58)
Birds	**1.60 (1.27-2.02)**	**1.59 (1.14-2.21)**	1.05 (0.75-1.46)	0.93 (0.51-1.70)	1.04 (0.74-1.46)
Farm animals	**1.52 (1.14-2.04)**	**2.23 (1.55-3.21)**	1.28 (0.87-1.89)	1.01 (0.47-2.15)	**1.57 (1.08-2.28)**
Other pets	**1.75 (1.41-2.16)**	**2.30 (1.76-3.01)**	**1.86 (1.45-2.39)**	1.42 (0.89-2.27)	**1.35 (1.01-1.81)**
***Children with allergic predisposition (n = 4,240)***					
Exposure to pets during pregnancy (ref: no)	0.89 (0.48-1.65)	1.08 (0.50-2.34)	1.17 (0.66-2.07)	0.98 (0.38-2.49)	**2.03 (1.16-3.53)**
Exposure to pets in the first years (ref: no)	1.09 (0.69-1.73)	**1.82 (1.06-3.12)**	0.94 (0.59-1.50)	1.28 (0.66-2.45)	**1.62 (1.02-2.57)**
Current exposure to pets (ref: no) †	**1.44 (1.17-1.78)**	**1.96 (1.52-2.53)**	1.20 (0.96-1.49)	0.81 (0.56-1.18)	0.92 (0.72-1.18)
Number and type of pets†					
1	**1.40 (1.10-1.77)**	**1.89 (1.42-2.51)**	1.21 (0.94-1.55)	0.97 (0.65-1.44)	0.92 (0.70-1.22)
≥2	**1.57 (1.10-2.25)**	**2.16 (1.43-3.29)**	1.17 (0.79-1.71)	0.41 (0.17-1.01)	0.92 (0.60-1.43)
Cats	**1.74 (1.22-2.46)**	**1.77 (1.15-2.74)**	1.21 (0.84-1.75)	0.70 (0.35-1.39)	0.75 (0.47-1.19)
Dogs	**1.53 (1.11-2.12)**	**2.37 (1.65-3.41)**	1.39 (0.99-1.93)	0.87 (0.48-1.57)	1.16 (0.80-1.68)
Birds	0.96 (0.58-1.60)	**1.89 (1.10-3.25)**	1.14 (0.70-1.87)	0.64 (0.26-1.58)	1.03 (0.62-1.72)
Farm animals	1.52 (0.94-2.48)	**2.08 (1.18-3.67)**	0.84 (0.46-1.54)	0.33 (0.10-1.35)	0.86 (0.47-1.57)
Other pets	1.37 (0.87-2.16)	**1.89 (1.10-3.25)**	1.19 (0.74-1.92)	1.16 (0.58-2.33)	0.80 (0.46-1.38)

^*^ Odds ratios (ORs) adjusted for age, gender, body mass index, breast feeding, use of domestic cooking and heating fuels, ETS, area per person, house decorations, parents education, avoidance behavior to pet exposure, commercial health insurance, districts and pet exposure variables.

†All ORs are computed to subjects with no current exposure to pets.

In the analysis stratified by gender (Table 3), among children without a family atopy history, in general girls had higher values of ORs than boys. For instance, significant associations of doctor-diagnosed asthma (OR =1.85, 95%CI: 1.23-2.79) and persistent phlegm (OR =1.82, 95%CI: 1.01-3.26) with pet exposure *in utero* were only observed in girls.

**Table 3:** Adjusted ORs (95%CI) of respiratory symptoms in relation to pet exposure in children without allergic predisposition (N = 26,809)^*^

**Characteristics**	**Persistent cough**	**Persistent phlegm**	**Doctor-diagnosed asthma**	**Current asthma**	**Current wheeze**
***Boys (n = 13,539)***					
Exposure to pets during pregnancy (ref: no)	1.07 (0.70-1.63)	1.38 (0.82-2.31)	1.10 (0.55-2.17)	**2.82 (1.60-4.95)**	**2.28 (1.50-3.45)**
Exposure to pets in the first years (ref: no)	1.08 (0.74-1.56)	1.12 (0.68-1.84)	**1.59 (1.09-2.33)**	**2.72 (1.62-4.57)**	**2.07 (1.41-3.03)**
Current exposure to pets (ref: no) †	**1.55 (1.33-1.80)**	**1.89 (1.55-2.31)**	**1.37 (1.14-1.64)**	1.17 (0.83-1.64)	**1.23 (1.01-1.51)**
Number and type of pets†					
1	**1.68 (1.43-1.97)**	**2.02 (1.64-2.49)**	**1.49 (1.23-1.81)**	1.30 (0.91-1.85)	1.24 (0.99-1.55)
≥2	0.99 (0.67-1.45)	1.34 (0.83-2.17)	0.83 (0.51-1.34)	0.59 (0.22-1.60)	1.20 (0.77-1.88)
Cats	**1.69 (1.31-2.19)**	**2.44 (1.80-3.32)**	**1.51 (1.11-2.06)**	1.49 (0.88-2.53)	1.29 (0.87-1.92)
Dogs	1.26 (0.96-1.65)	1.32 (0.91-1.92)	1.11 (0.80-1.54)	0.73 (0.37-1.51)	1.12 (0.78-1.61)
Birds	**1.54 (1.10-2.14)**	1.56 (0.98-2.48)	1.08 (0.69-1.68)	0.76 (0.31-1.85)	0.76 (0.45-1.29)
Farm animals	1.55 (1.00-2.39)	1.49 (0.80-2.79)	0.93 (0.50-1.72)	0.88 (0.28-2.81)	0.86 (0.43-1.71)
Other pets	**1.44 (1.05-1.97)**	**2.33 (1.62-3.35)**	**1.79 (1.28-2.50)**	1.69 (0.96-3.00)	**1.59 (1.10-2.29)**
***Girls (n = 13,270)***					
Exposure to pets during pregnancy (ref: no)	1.13 (0.70-1.84)	**1.82 (1.01-3.26)**	**1.85 (1.23-2.79)**	**3.16 (1.42-7.04)**	**2.26 (1.27-4.02)**
Exposure to pets in the first year (ref: no)	1.15 (0.79-1.67)	1.10 (0.64-1.89)	**1.60 (1.02-2.53)**	**3.81 (2.05-7.07)**	**2.76 (1.79-4.26)**
Current exposure to pets (ref: no)^†^	**1.65 (1.42-1.92)**	**1.89 (1.53-2.34)**	**1.58 (1.29-1.93)**	1.15 (0.78-1.70)	**1.37 (1.11-1.70)**
Number and type of pets†					
1	**1.65 (1.40-1.94)**	**1.83 (1.45-2.30)**	**1.62 (1.31-2.01)**	1.23 (0.81-1.85)	**1.39 (1.11-1.75)**
≥2	**1.66 (1.21-2.28)**	**2.20 (1.46-3.33)**	1.40 (0.91-2.17)	0.78 (0.29-2.14)	1.28 (0.79-2.07)
Cats	**1.88 (1.46-2.42)**	**2.27 (1.63-3.18)**	**1.94 (1.40-2.69)**	1.47 (0.76-2.83)	**1.44 (1.02-2.04)**
Dogs	1.24 (0.95-1.63)	1.33 (0.91-1.96)	**1.42 (1.02-1.98)**	0.85 (0.41-1.77)	1.35 (0.95-1.94)
Birds	**1.67 (1.21-2.30)**	**1.61 (1.00-2.60)**	1.00 (0.60-1.67)	1.14 (0.50-2.59)	1.37 (0.88-2.14)
Farm animals	**1.52 (1.02-2.26)**	**2.92 (1.86-4.58)**	**1.67 (1.02-2.73)**	1.14 (0.42-3.14)	**2.34 (1.49-3.68)**
Other pets	**2.09 (1.57-2.79)**	**2.26 (1.52-3.36)**	**1.97 (1.35-2.87)**	1.06 (0.47-2.43)	1.08 (0.67-1.74)

^*^Odds ratios (ORs) adjusted for age, body mass index, breast feeding, use of domestic cooking and heating fuels, ETS, area per person, house decorations, parents education, avoidance behavior to pet exposure, commercial health insurance, districts and pet exposure variables.

†All ORs are computed to subjects with no current exposure to pets.

Among children with family atopy history, in general girls had higher values of ORs than boys (Table 4). More significant associations were observed in girls than in boys. For example, in girls, significant associations were observed between doctor-diagnosed asthma and pet exposure during pregnancy (ORs = 2.03; 95%CI: 1.01-4.33) and current pet exposure (ORs = 1.37; 95%CI: 1.00-1.88), and between current asthma and pet exposure in the first year of life (ORs = 2.36; 95% CI: 1.08-5.16) only in girls. However, in boys, significant associations were only observed between pet exposure and persistent phlegm.

**Table 4:** Adjusted ORs (95%CI) of respiratory symptoms in relation to pet exposure in children with allergic predisposition (N = 4,240)^*^

**Characteristics**	**Persistent cough**	**Persistent phlegm**	**Doctor-diagnosed asthma**	**Current asthma**	**Current wheeze**
***Boys (n = 2,134)***					
Exposure to pets during pregnancy (ref: no)	0.80 (0.32-2.00)	1.40 (0.52-3.77)	0.70 (0.29-1.70)	0.31 (0.04-2.26)	1.76 (0.81-3.82)
Exposure to pets in the first years (ref: no)	0.77 (0.40-1.49)	1.26 (0.58-2.72)	0.78 (0.37-1.62)	0.42 (0.10-1.77)	1.30 (0.63-2.67)
Current exposure to pets (ref: no) †	1.26 (0.93-1.71)	**1.74 (1.20-2.51)**	1.07 (0.79-1.45)	0.70 (0.42-1.18)	0.89 (0.63-1.26)
Number and type of pets†					
1	1.24 (0.88-1.76)	**1.75 (1.16-2.65)**	1.10 (0.78-1.54)	0.87 (0.50-1.50)	0.88 (0.59-1.31)
≥2	1.32 (0.77-2.27)	1.68 (0.88-3.21)	0.99 (0.57-1.74)	0.28 (0.07-1.15)	0.92 (0.50-1.69)
Cats	1.43 (0.86-2.40)	1.82 (0.99-3.33)	1.02 (0.60-1.71)	0.13 (0.04-1.01)	0.57 (0.28-1.14)
Dogs	1.21 (0.74-1.97)	1.72 (0.97-3.04)	1.23 (0.78-1.96)	0.66 (0.28-1.55)	1.19 (0.71-2.00)
Birds	0.70 (0.32-1.57)	1.40 (0.59-3.32)	1.30 (0.68-2.48)	1.18 (0.46-3.01)	1.13 (0.56-2.27)
Farm animals	1.13 (0.50-2.57)	1.33 (0.49-3.62)	0.68 (0.26-1.77)	0.32 (0.04-2.37)	0.65 (0.22-1.90)
Other pets	1.70 (0.94-3.06)	**2.45 (1.25-4.82)**	1.11 (0.59-2.10)	1.59 (0.71-3.57)	1.04 (0.52-2.08)
***Girls (n = 2,106)***					
Exposure to pets during pregnancy (ref: no)	1.02 (0.44-2.37)	0.77 (0.23-2.66)	**2.03 (1.01-4.33)**	1.98 (0.66-5.96)	**2.48 (1.11-5.56)**
Exposure to pets in the first years (ref: no)	1.64 (0.85-3.16)	**2.91 (1.36-6.22)**	1.08 (0.58-2.01)	**2.36 (1.08-5.16)**	**2.01 (1.09-3.69)**
Current exposure to pets (ref: no) †	**1.66 (1.24-2.22)**	**2.23 (1.56-3.17)**	**1.37 (1.00-1.88)**	0.92 (0.54-1.58)	0.98 (0.69-1.38)
Number and type of pets†					
1	**1.59 (1.14-2.21)**	**2.06 (1.37-3.08)**	1.36 (0.95-1.95)	1.06 (0.59-1.89)	0.99 (0.67-1.47)
≥2	**1.87 (1.15-3.03)**	**2.70 (1.55-4.71)**	1.41 (0.82-2.40)	0.57 (0.18-1.86)	0.95 (0.50-1.77)
Cats	**2.09 (1.29-3.40)**	1.71 (0.91-3.23)	1.45 (0.86-2.44)	1.58 (0.73-3.43)	0.98 (0.53-1.83)
Dogs	**2.01 (1.29-3.11)**	**3.20 (1.97-5.19)**	**1.63 (1.02-2.61)**	1.04 (0.46-2.34)	1.15 (0.68-1.95)
Birds	1.27 (0.65-2.49)	**2.54 (1.25-5.16)**	1.03 (0.48-2.19)	---‡	0.97 (0.45-2.10)
Farm animals	**1.92 (1.04-3.52)**	**2.82 (1.41-5.64)**	1.04 (0.49-2.23)	0.30 (0.07-2.21)	1.09 (0.53-2.28)
Other pets	1.03 (0.50-2.14)	1.25 (0.49-3.20)	1.30 (0.63-2.68)	0.60 (0.14-2.51)	0.54 (0.21-1.37)

^*^Odds ratios (ORs) adjusted for age, body mass index, breast feeding, use of domestic cooking and heating fuels, ETS, area per person, house decorations, parents education, avoidance behavior to pet exposure, commercial health insurance, districts and pet exposure variables.

^†^All ORs are computed to subjects with no current exposure to pets. ‡Estimate not available due to small number of exposed cases.

## Discussion

In this study, both prenatal and postnatal pet exposure were significantly associated with respiratory symptoms, and children without a familial predisposition were found to be more susceptible to respiratory symptoms and diseases caused by pet exposure than those with a genetic predisposition. This indicates that pet exposure may be an important causal risk factor for non-allergic asthma, a condition that deserves more attention.

There has been little literature about the modification effects of allergic predisposition on the association between pet exposure and respiratory symptoms and asthma in children. This study may serve as a basis of the research and facilitate future studies in this regard. There have been reports that children without an allergic predisposition may be more susceptible to certain environmental pollutants (such as environmental tobacco smoke [ETS]) than children with an allergic predisposition [22-24]. For example, in a study of 5,762 school-aged children in 12 Southern California communities, the effects of involuntary tobacco smoke exposure on wheeze were largest in children without a family history of asthma or a family history of atopy, and *in utero* exposure to ETS was positively associated with doctor-diagnosed asthma only in children without a family history of asthma (OR = 1.9, 95% CI, 1.0–3.7) [22]. Results from the European Community Respiratory Health Survey (ECRHS) also showed that the associations of maternal smoking with wheeze (OR = 1.23, 95% CI, 1.08–1.40), asthma symptom (OR = 1.24, 95% CI, 1.06–1.44), and chronic bronchitis (OR = 1.21, 95% CI, 1.01–1.44) were only significant in non-atopic individuals [23]. However, assessing the association between pet exposure and respiratory diseases in children with an allergic predisposition has been difficult due to the lack of reliable and readily available data, which is possibly a result of the fact that parents tend to take allergen-avoidance measures when they or their child are diagnosed with allergies. For example, a Prevention and Incidence of Asthma and Mite Allergy (PIAMA) study reported that allergic parents took allergen-avoidance measures more often than did non-allergic parents [25]. In a study from Sweden, parents often gave up pets if their child developed asthma or allergic symptoms [26]. Results from the 12 European birth cohort study also showed that families with allergies were less likely to keep a pet at home [8]. The data collected for the present study may be considered more reliable and less biased due to the population’s lack of knowledge of the triggers of asthma, as is evident in our study where 18.5% of the participants with an allergic predisposition lived in a family with pets, as compared to the significantly smaller percentage (15.4%) of the participants without an allergic predisposition (*χ*^2^ = 26.23, *p* <0.001), and only 0.8% of families gave up pets because of the allergic illness in the family.

Tables 3 and 4 seemed to indicate that, among all pets, cats were associated with the highest ORs of having respiratory symptoms in children. There have been other studies that seem to be supportive of this finding. For example, the studies of Finnish and Russian children reported that continuous home exposure to cat allergens increased the risk of self-reported allergic asthma, whereas exposure to dogs decreased such risk [14]. Oberle *et al.* found a significant association between continuous cat exposure from early life onwards and asthma in childhood, but found that exposure to dogs was not related to the prevalence of asthma [27]. There also have been reports that cat allergens could be more potent sensitizers than dog allergens [28]. The major cat allergen is Fel d1, a type of secretoglobin; whereas the major dog allergen is Can f1, a type of lipocalin. These two major allergens present different biochemical and pathogenic characteristics [29,30].

The present study showed that boys and girls differed in the association between pet exposure and respiratory symptoms/illnesses (Table 3 and Table 4); in particular, girls appeared to be more susceptible to respiratory symptoms/illnesses than boys. However, because there has been little literature on the gender-specific effect on the association between pet exposure and respiratory symptoms/illnesses among children, we conjecture that there are several possible reasons for such difference. The first reason lies in the fact that males and females respond differently to exposure to environmental factors due to the differences in their airways from fetal life to adult life, with females having smaller lungs and so having slightly greater airway reactivity than males [15,16,31,32]. Second, non-allergic asthmatics tend to have higher nasal and bronchial epithelia sensibility to stimuli such as environmental pollutants, strong smells, cold air, wind or respiratory viruses than allergic asthmatics [33]. Inouye *et al.* reported that the clinical history of atopy appeared to be significantly more frequent in allergic asthmatics, and hay fever appeared to most effective in providing a protective effect against the development of non-allergic asthma [34]. Furthermore, Romanet-Manent *et al.* also reported that the female sex is associated with an increasing risk of non-allergic asthma, compared to allergic asthma [33]. These reported findings are consistent with our findings from this study, where 19.6% of the girls were found to ever have allergies and 8.9% of the girls were found to ever have hay fever, as compared to the significantly higher percentages in males (21.7% and 13.4% respectively). Therefore, we conjecture that, among children with an allergic predisposition, girls may be more likely to develop non-allergic asthma and therefore may be more sensitive to pet exposure than boys. Third, it has been reported that the deposition of particles in the lung varies by gender, with greater lung deposition fractions of particles being in all regions of the lung in females [35,36]. Sunyer *et al.* suggested that females and males had different deposition patterns, which might be partly accountable for the difference in their responses to particles [37]. Therefore, we conjecture that girls may be more susceptible to the indoor dust carrying over mite, pet allergens, and endotoxin than boys [38]. The last reason for the gender-related difference observed in this study may be partly attributable to the combination of China’s “one child” policy and its long tradition of strong preference for males that have put males at an advantage by getting better healthcare than females [39]. It is well known that the one-child policy can alter the number and gender composition of children who become part of family, and in turn these family composition characteristics will alter couple’s abilities or desires to provide quality care to individual children. Furthermore, because China is characterized by son preference which often affects the care received by young children, boys receive better child care, food, and health care than girls. For instance, approximately 25.9% of the males had commercial or social health insurance whereas only 14.7% of the females had commercial health insurance (Table 1). However, much still remains unknown about why boys and girls respond differently to pet exposure; therefore, further investigation is required to determine the cause of such difference and new methods are needed for this purpose.

Our study has the following limitations. First, we have studied respiratory prevalence, rather than respiratory symptom incidence, which is known to be prone to be affected by disease duration rather than disease incidence. Second, we cannot establish a temporal relationship between exposure and the outcome from a cross-sectional study. On the other hand, we must also acknowledge that large epidemiological studies of asthma often rely on the self-reported symptom history and physician’s diagnosis of asthma for they are generally easier, faster and less expensive to access. Finally, the standardized skin testing to aeroallergens was not required for the present study, which may effect interpretation of findings related to allergic *versus* non-allergic status.

## Conclusions

This study showed that pet exposure was associated with increases susceptibility to respiratory symptoms/diseases in children, with girls being more susceptible than boys, and that children without a genetic predisposition appeared to be more susceptible than those with a genetic predisposition. This indicates that pet exposure may be an important causal factor for non-allergic asthma.

## Abbreviations

SNECC, Seven Northeast Cities of China; OR, Odds ratio; 95%CI, 95% Confidence interval; SD, Standard deviation; ETS, Environmental tobacco smoke.

## Competing interest

Authors report no conflicts of interest. Authors are alone responsible for the content and writing of the paper.

## Authors’ contributions

Conceived and designed the experiments: GHD YLL. Performed the experiments: GHD. Analyzed the data: GHD YLL JW. Contributed reagents/materials/analysis tools: GHD MML DW WHR YDZ. Wrote the paper: GHD YLL JW. Revised the paper: GHD YLL JW. Contributed the investigation: MML DW WHR YDZ. All authors read and approved the final manuscript.

## References

[B1] Lombardi E Simoni M La Grutta S Viegi G Bisanti L Chellini E Dell’Orco V Migliore E Petronio MG Pistelli R Rusconi F Sestini P Forastiere F Galassi C SIDRIA-2 Collaborative Group Effects of pet exposure in the first year of life on respiratory and allergic symptoms in 7-yr-old children. The SIDRIA-2 study Pediatr Allergy Immunol 2010 21 268 276 20444167 10.1111/j.1399-3038.2009.00910.x

[B2] Behrens T Maziak W Weiland SK Rzehak P Siebert E Keil U Symptoms of asthma and the home environment. The ISAAC I and III cross-sectional surveys in Münster, Germany Int Arch Allergy Immunol 2005 137 53 61 15785082 10.1159/000084613

[B3] Brunekreef B Von Mutius E Wong GK Odhiambo JA Clayton TO the ISAAC Phase Three Study Group: Early life exposure to farm animals and symptoms of asthma, rhinoconjunctivitis and eczema: an ISAAC Phase Three Study Int J Epidemiol 2012 10.1093/ije/dyr216 22287135

[B4] Karimi M Mirzaei M Baghiani Moghadam B Fotouhi E Zare Mehrjardi A Pet exposure and the symptoms of asthma, allergic rhinitis and eczema in 6–7 years old children Iran J Allergy Asthma Immunol 2011 10 123 127 21625021

[B5] Almqvist C Garden F Kemp AS Li Q Crisafulli D Tovey ER Xuan W Marks GB CAPS Investigators: Effects of early cat or dog ownership on sensitisation and asthma in a high-risk cohort without disease-related modification of exposure Paediatr Perinat Epidemiol 2010 24 171 178 20415774 10.1111/j.1365-3016.2010.01095.x

[B6] Bufford JD Gern JE Early exposure to pets: good or bad? Curr Allergy Asthma Rep 2007 7 375 382 17697647 10.1007/s11882-007-0057-4

[B7] Apelberg BJ Aoki Y Jaakkola JJ Systematic review: Exposure to pets and risk of asthma and asthma-like symptoms J Allergy Clin Immunol 2001 107 455 460 11240945 10.1067/mai.2001.113240

[B8] Chen Y Rennie D Cormier Y McDuffie H Pahwa P Dosman J Reduced Risk of Atopic Sensitization among Farmers: The Humboldt Study Int Arch Allergy Immunol 2007 144 338 342 17664888 10.1159/000106460

[B9] Dimich-Ward H Chow Y Chung J Trask C Contact with livestock–a protective effect against allergies and asthma? Clin Exp Allergy 2006 36 1122 1129 16961711 10.1111/j.1365-2222.2006.02556.x

[B10] Eller E Roll S Chen CM Herbarth O Wichmann HE von Berg A Krämer U Mommers M Thijs C Wijga A Brunekreef B Fantini MP Bravi F Forastiere F Porta D Sunyer J Torrent M Høst A Halken S Lødrup Carlsen KC Carlsen KH Wickman M Kull I Wahn U Willich SN Lau S Keil T Heinrich J Working Group of GA2LEN–Work Package 1.5 Birth Cohorts: Meta-analysis of determinants for pet ownership in 12 European birth cohorts on asthma and allergies: a GA2LEN initiative Allergy 2008 63 1491 1498 18721248 10.1111/j.1398-9995.2008.01790.x

[B11] Lodge CJ Allen KJ Lowe AJ Hill DJ Hosking CS Abramson MJ Dharmage SC Perinatal cat and dog exposure and the risk of asthma and allergy in the urban environment: a systematic review of longitudinal studies Clin Dev Immunol 2012 2012 176484 22235226 10.1155/2012/176484 PMC3251799

[B12] Bertelsen RJ Carlsen KC Granum B Carlsen KH Håland G Devulapalli CS Munthe-Kaas MC Mowinckel P Løvik M Do allergic families avoid keeping furry pets? Indoor Air 2010 20 187 195 20158528 10.1111/j.1600-0668.2009.00640.x

[B13] Roy A Wisnivesky JP Racial and ethnic differences in the use of environmental control practices among children with asthma J Asthma 2010 47 507 512 20536284 10.3109/02770901003734314

[B14] Hugg TT Jaakkola MS Ruotsalainen R Pushkarev V Jaakkola JJ Exposure to animals and the risk of allergic asthma: a population-based cross-sectional study in Finnish and Russian children Environ Health 2008 7 28 18538018 10.1186/1476-069X-7-28 PMC2430194

[B15] Clougherty JE A growing role for gender analysis in air pollution epidemiology Environ Health Perspect 2010 118 167 176 20123621 10.1289/ehp.0900994 PMC2831913

[B16] Lodrup Carlsen KC Lovik M Granum B Mowinckel P Carlsen KH Soluble CD14 at 2 yr of age: gender-related effects of tobacco smoke exposure, recurrent infections and atopic diseases Pediatr Allergy Immunol 2006 17 304 312 16771785 10.1111/j.1399-3038.2006.00412.x

[B17] Dong GH Ma YN Ding HL Jin J Cao Y Zhao YD He QC Housing characteristics, home environmental factors and respiratory health in 3945 pre-school children in China Int J Environ Health Res 2008 18 267 282 18668415 10.1080/09603120701842864

[B18] Downes MJ Roy A McGinn TG Wisnivesky JP Factors associated with furry pet ownership among patients with asthma J Asthma 2010 47 742 749 20684732 10.3109/02770903.2010.491146

[B19] Salo PM Xia J Johnson CA Li Y Avol EL Gong J London SJ Indoor allergens, asthma, and asthma-related symptoms among adolescents in Wuhan, China Ann Epidemiol 2004 14 543 550 15350953 10.1016/j.annepidem.2003.09.015 PMC1626161

[B20] Zhang JJ Hu W Wei F Wu G Korn LR Chapman RS Children’s Respiratory Morbidity Prevalence in Relation to Air Pollution in Four Chinese Cities Environ Health Perspect 2002 110 961 967 12204833 10.1289/ehp.02110961 PMC1240998

[B21] Peters JM Avol E Navidi W London SJ Gauderman WJ Lurmann F Linn WS Margolis H Rappaport E Gong H Thomas DC A study of twelve Southern California communities with differing levels and types of air pollution. I. Prevalence of respiratory morbidity Am J Respir Crit Care Med 1999 159 760 767 10051248 10.1164/ajrccm.159.3.9804143

[B22] Gilliland FD Li YF Peters JM Effects of maternal smoking during pregnancy and environmental tobacco smoke on asthma and wheezing in children Am J Respir Crit Care Med 2001 163 429 436 11179118 10.1164/ajrccm.163.2.2006009

[B23] Svanes C Omenaas E Jarvis D Gulsvik A Burney P Parental smoking in childhood and adult obstructive lung disease: results from the European Community Respiratory Health Survey Thorax 2004 59 295 302 15047948 10.1136/thx.2003.009746 PMC1763798

[B24] Dong GH Wang D Yang ZH Zhang PF Ren WH Zhao YD He QC Gender-specific differences in effects of prenatal and postnatal environmental tobacco smoke exposure on respiratory symptoms in 23,474 children with and without allergic predisposition: results from 25 districts of northeast China Int J Environ Health Res 2011 21 173 188 21547813 10.1080/09603123.2010.515673

[B25] van Strien RT Koopman LP Kerkhof M Spithoven J de Jongste JC Gerritsen J Neijens HJ Aalberse RC Smit HA Brunekreef B Mite and pet allergen levels in homes of children born to allergic and nonallergic parents: the PIAMA study Environ Health Perspect 2002 110 A693 A698 12417497 10.1289/ehp.021100693 PMC1241089

[B26] Bornehag CG Sundell J Hagerhed L Janson S DBH Study Group: Pet-keeping in early childhood and airway, nose and skin symptoms later in life Allergy 2003 58 939 944 12911425 10.1034/j.1398-9995.2003.00050.x

[B27] Oberle D von Mutius E von Kries R Childhood asthma and continuous exposure to cats since the first year of life with cats allowed in the child’s bedroom Allergy 2003 58 1033 1036 14510722 10.1034/j.1398-9995.2003.00285.x

[B28] Murray AB Ferguson AC Morrison BJ The frequency and severity of cat allergy vs. dog allergy in atopic children J Allergy Clin Immunol 1983 72 145 149 6886252 10.1016/0091-6749(83)90522-5

[B29] Virtanen T Lipocalin allergens Allergy 2001 56 48 51 11298009 10.1034/j.1398-9995.2001.00915.x

[B30] Kaiser L Gro¨nlund H Sandalova T Ljunggren HG van Hage-Hamsten M Achour A Schneider G The crystal structure of the major cat allergen Fel d 1, a member of the secretoglobin family J Biol Chem 2003 278 37730 37735 12851385 10.1074/jbc.M304740200

[B31] Becklake MR Kauffmann F Gender differences in airway behaviour over the human life span Thorax 1999 54 1119 1138 10567633 10.1136/thx.54.12.1119 PMC1763756

[B32] Yunginger JW Reed CE O’Connell EJ Melton LJ O’Fallon WM Silverstein MD A community-based study of the epidemiology of asthma. Incidence rates, 1964–1983 Am Rev Respir Dis 1992 146 888 894 1416415 10.1164/ajrccm/146.4.888

[B33] Romanet-Manent S Charpin D Magnan A Lanteaume A Vervloet D the EGEA Cooperative Group: Allergic vs nonallergic asthma: what makes the difference? Allergy 2002 57 607 613 12100301 10.1034/j.1398-9995.2002.23504.x

[B34] Inouye T Tarlo S Broder I Corey P Davies G Leznoff A Mintz S Thomas P Severity of asthma in skin test-negative and skin test-positive patients J Allergy Clin Immunol 1985 75 313 319 3968339 10.1016/0091-6749(85)90063-6

[B35] Kim CS Hu SC Total respiratory tract deposition of fine micrometer-sized particles in healthy adults: empirical equations for sex and breathing pattern J Appl Physiol 2006 101 401 412 16849812 10.1152/japplphysiol.00026.2006

[B36] Kohlhäufl M Brand P Scheuch G Meyer TS Schulz H Häussinger K Heyder J Increased fine particle deposition in women with asymptomatic nonspecific airway hyperresponsiveness Am J Respir Crit Care Med 1999 159 902 906 10051270 10.1164/ajrccm.159.3.9805036

[B37] Sunyer J Schwartz J Tobias A Macfarlane D Garcia J Anto JM Patients with chronic obstructive pulmonary disease are at increased risk of death associated with urban particle air pollution: a case-crossover analysis Am J Epidemiol 2000 151 50 56 10625173 10.1093/oxfordjournals.aje.a010121

[B38] Gent JF Belanger K Triche EW Bracken MB Beckett WS Leaderer BP Association of pediatric asthma severity with exposure to common household dust allergens Environ Res 2009 109 768 774 19473655 10.1016/j.envres.2009.04.010 PMC2706291

[B39] Shi Z Lien N Kumar BN Dalen I Holmboe-Ottesen G The sociodemographic correlates of nutritional status of school adolescents in Jiangsu Province, China J Adolesc Health 2005 37 313 322 16182142 10.1016/j.jadohealth.2004.10.013

